# A New Work on Parturition

**Published:** 1849-01

**Authors:** 


A NEW WORK ON PARTURITION.

Professor Miller is about publishing a work on the Process of Parturition. It is already in press, and will be issued by J. V. Cowling, publisher, in February. The numerous pupils and friends of the author will be gratified at this announcement.

